# Observation of the transition from capacitive to inductive synaptic behavior

**DOI:** 10.1093/nsr/nwag278

**Published:** 2026-05-18

**Authors:** Juan Bisquert

**Affiliations:** Instituto de Tecnología Química (Universitat Politècnica de València-Agencia Estatal Consejo Superior de Investigaciones Científicas), Spain

The search for suitable materials for ‘synapses’ in physical computing focuses on bridging the gap between digital logic and the physical world. Unlike traditional computing, physical computing relies on materials that can sense, respond, and adapt to environmental inputs such as touch, light, pressure, or temperature. In this context, ‘synapses’ refer to the connections and mechanisms—often inspired by biological systems—that enable interaction, signal transmission, and learning within hardware systems. Exploring conductive fabrics, smart polymers, bio-inspired components, and neuromorphic elements allows designers and engineers to create more responsive, adaptive, and interactive systems, expanding the possibilities of how humans and machines interact.

**Figure 1.**
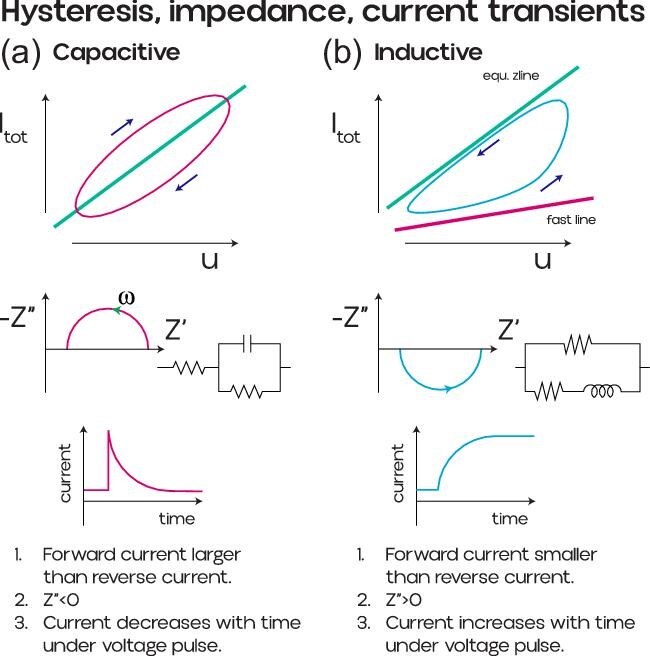
Scheme of (a) capacitive and (b) inductive hysteresis, and the related impedance and transient current response. Reproduced from [3].

A synapse, normally formed by an electrical memristor, is an object that shows plasticity, where some voltage pulses increase the conductance (in potentiation), and opposite voltage pulses decrease it (in depression). Recently, the physical behavior of these systems has been comprehensively studied, revealing a clear interrelation between different measurable responses [1–3], particularly the cyclic voltage characteristics exhibiting pronounced hysteresis and the impedance spectra obtained under small alternating current perturbations, with a main distinction between capacitive (Fig. 1a) and inductive (Fig. 1b) hysteresis.

Potentiation, i.e. the increase of conductance, is naturally associated with inductive behavior, since an inductor is, by definition, an element in which the current (and thus effective conductance) increases with time under an applied voltage. However, the conventional electromagnetic inductor is not typically present in synaptic elements based on ionic-electronic conductors. Instead, the observed delay originates from specific physicochemical processes, commonly described as a chemical inductor [4].

On the other hand, depression, the decrease of conductance, is associated with a capacitive response. This is less intuitive, as an ideal capacitor does not conduct. In synaptic systems, however, one can define a ‘conduction capacitance’ in which the same underlying mechanism responsible for potentiation (the chemical inductor) effectively acquires a negative contribution, leading to a progressive reduction of conductance [5].

This general framework has been well established and experimentally demonstrated in a variety of systems, including halide perovskite memristors [6] and asymmetric electrolytic nanochannel memristors [7]. However, previous studies have largely relied on phenomenological descriptions based on a small set of differential equations, such as the conductance activated quasi-linear memristor model [8], while a microscopic understanding of the mechanisms that distinguish capacitive from inductive behavior has remained elusive.

In a new work published in *National Science Review*, Zhang *et al*. [9] provide an advance of this picture by establishing a microscopic and physically grounded mechanism that links ionic transport states to the emergence of capacitive and inductive hysteresis within a single symmetrical nanofluidic synaptic element. Specifically, they demonstrate that the transition between depression- and potentiation-like behaviors is governed by the relationship between interionic distance and the Bjerrum length, enabling a concentration-dependent and ion-species-dependent switching between capacitive (double layer-dominated) and inductive (ion-pairing-driven) regimes, as shown in Fig. 3 of the paper [9]. Moreover, their system achieves programmable synaptic plasticity without structural modification, where facilitation or depression can be selected simply by changing the ionic species, and they further integrate these effects to realize functional ionic circuits (e.g. tunable high-pass filters), thereby bridging phenomenological descriptions with a unified microscopic understanding of neuromorphic iontronic devices.

Overall, this work represents a significant step toward a unified, physically grounded framework for synaptic functionality in iontronic systems, opening new avenues for the rational design of adaptive, multifunctional elements in neuromorphic and physical computing.
